# Maternal HPV-antibodies and seroconversion to HPV in children during the first 3 years of life

**DOI:** 10.1038/s41598-022-06343-z

**Published:** 2022-02-09

**Authors:** Stina Syrjänen, Tim Waterboer, Marjut Rintala, Michael Pawlita, Kari Syrjänen, Karolina Louvanto, Seija Grenman

**Affiliations:** 1 Department of Oral Pathology, Institute of Dentistry, Faculty of Medicine, University of Turku, Lemminkäisenkatu 2, 20520 Turku, Finland; 2 Department of Pathology, University of Turku, Turku University Hospital, Turku, Finland; 3 Department of Genome Modifications and Carcinogenesis, Infection and Cancer Program, German Cancer Research Center (DKFZ), Heidelberg, Germany; 4 Department of Obstetrics and Gynecology, University of Turku, Turku University Hospital, Turku, Finland; 5 Department of Clinical Research, Biohit Oyj, Helsinki, Finland; 6 Department of Obstetrics and Gynecology, Tampere University and Tampere University Hospital, Tampere, Finland

**Keywords:** Immunology, Medical research

## Abstract

To assess the dynamics of human papillomavirus (HPV) serology, we analyzed HPV6-,11-,16-,18-, and 45 antibodies in infants during the first 36 months of their life. Serial serum samples of 276/327 mother–child pairs were collected at baseline (mothers) and at months 1, 2, 6, 12, 24 and 36 (offspring), and tested for HPVL1-antibodies using the GST-L1 assay. Concordance between maternal and infant HPV-antibody levels remained high until month-6 (p <  = 0.001), indicating maternal antibody transfer. At 1 month, 40–62% of the infants tested seropositive to any of the 5 HPV-types. Between 1–3 years of age, 53% (58/109) of the children born to HPV-seronegative mothers tested HPV-seropositive. Times to positive seroconversion varied between13.4 and 18.7 months, and times to negative seroconversion (decay) between 8.5 and 9.9 months. Significant independent predictors of infants’ seroconversion to LR-HPV were hand warts and mother’s history of oral warts and seroconversion to LR-HPV. No predictors of seroconversion to HR-HPV were identified. Maternal HPV-IgG-antibodies are transferred to her offspring and remain detectable for 6 months, corroborating the IgG molecule’s half-life. Seroconversion to HPV-genotypes 6, 11, 16 and 18 was confirmed among children born to HPV-seronegative mothers, implicating an immune response to these HPV-genotypes during early infancy.

## Introduction

Human papillomavirus (HPV)-infection has been traditionally considered a sexually transmitted infection (STI). However, HPV DNA has been found in oral and genital samples of neonates and young children as well as in the placenta and cord blood, suggesting the possibility of maternal transmission^1–6^. Meta-analysis on 3128 mother–child pairs showed that children of HPV-positive mothers were 33% more likely to be HPV-positive than children born to HPV-negative mothers^5^. Another systematic review confirmed intrauterine HPV-transmission, with a pooled frequency of 4.9% for antenatal vertical HPV transmission^6^.

Only few studies have focused on HPV-serology in young children and on maternal HPV-antibodies in early infancy^7–11^. During the era of HPV-vaccinations, it is important to understand the dynamics of maternal HPV-immunization and its impact on the mother-to-child HPV-transmission and HPV-burden in young children.

As a part of the prospective Finnish Family HPV-Study, we evaluated the dynamics of HPV-type-specific antibodies in the offspring, with special reference to maternal HPV-antibodies and infants’ seroconversion to the L1 protein of HPV 6/11/16/18/45 during the first 3 years of their life.

## Methods

### Study design and population

The Finnish Family HPV-Study (FFHPV) is a longitudinal cohort designed to explore the dynamics of HPV-infection in the enrolled mothers, fathers and their offspring. Between 1998–2001, a total of 327 pregnant (3rd trimester) women and their spouses were enrolled at Maternity Unit of Turku University Hospital, (Finland), as previously described^1,12^. The study design was approved by the Research Ethics Committee of Turku University Hospital (#3/1998), with subsequent amendments (#2/2006 and 45/180/2010), and all methods were performed in accordance with the relevant guidelines and regulations. Written informed consent was obtained from all parents who agreed to participate in the study. The mothers’ demographic data as well as their oral and genital HPV-status at baseline have been detailed elsewhere^1,12–15^.

### HPV-serology

Blood samples were taken from all 327 pregnant women at baseline, and at 12-, 24- and 36-month follow-up (FU) visits, as described earlier^13^. Only the baseline HPV-serology of the mothers was included in the present analysis. From the infants, the blood samples were taken at 1-, 2-, 6-,12-, 24- and 36-month FU visits (all tested children irrespective of the mother’s serostatus), including 232, 237, 263, 272, 268 and 243 children, respectively. The first blood sample of the newborn was taken from the head, while the subsequent samples were drawn from the arm bend. The actual timing (mean months and range) of the FU visits was as follows: 1.2 (0.5–1.9), 2.2 (1.6–3.8), 6.4 (5.2–9.4), 12.6 (10.4–15.5), 24.8 (21.4–32.9), and 36.9 (34.1–47.5) months, respectively.

After collection, the blood samples were centrifuged at 2400 rpm for 10 min (Sorvall GLC-2, DuPont Instrument, Newtown, Connecticut, USA), the serum was divided into three aliquots, and stored first at − 20 °C for no longer than 1 week, and then at − 70 °C until delivered to the German Cancer Research Center (DKFZ), Heidelberg, Germany for serological analysis.

Antibodies to the major capsid protein L1 of HPV types 6/11/16/18/45 were analyzed by multiplex HPV serology, based on glutathione S-transferase fusion protein capture to fluorescent beads, as previously described^16^. Sera were scored as positive when the antigen-specific median fluorescence intensity (MFI) values were higher than the cut-off level of 200 MFI for the L1 antigen of individual HPV-genotypes^13,16^. Seroconversion event at any FU visit was recorded if both of the following conditions apply: (1) at least twofold increase of the previous MFI-value, and reaching at least the cut-off level (MFI 200) (2) any MFI-value exceeding the 200 MFI cut-off level as compared with the baseline status (i.e., the 1-month visit). Similarly, antibody decay was defined by the same two conditions in reverse order: (1) at least twofold decrease of the previous MFI-value, and (2) fall of the MFI-value below the single cut-off level of 200 MFI.

### Statistical analysis

Scatterplots were generated to show the correlation between the maternal and infant serum HPV antibody-levels. The bivariate correlation of the HPV-antibody-levels between the maternal sera (baseline) and her offspring sera taken at 1-, 6-, and 12-month visit was tested using the Spearman r correlation coefficients, separately for the five HPV types.

Frequency tables were analyzed using the χ^2^-test, with the likelihood ratio or Fisher's exact test for categorical variables. Differences in the means of continuous variables were analyzed using Mann–Whitney’s test or Kruskal–Wallis’s test for two and multiple independent samples, respectively. Univariate survival analysis for serological outcomes (seroconversion, decay) was based on the Kaplan–Meier method, with log-rank statistics.

Co-variates of seroprevalance and seroconversion were analysed using generalized estimating equation (GEE) model, stratified by the five HPV types. In this analysis, we assumed that HPV-seroconversion depends on time since the previous sample, and a time variable was included as a covariate in these GEE models^15^, We entered into multivariate GEE models several covariates previously confirmed or implicated as risk factors of HPV-infections in our cohort^12,14,15^. SPSS^®^ for windows, version 26 (IBM Corp. Armonk, NY) and STATA (STATA/SE 15.1, Stata Corp., College Station, TX, USA) were used, all tests being performed as two-sided, with p < 0.05 as the level of significance.

### Ethics approval

Approval was granted by the Research Ethics Committee of Turku University Hospital (#3/1998), with subsequent amendments (#2/2006 and 45/180/2010).

### Consent to participate

Informed consent (in written) was obtained from all mothers- and fathers-to-come who agreed to participate in the FFHPV cohort.

### Consent for publication

All authors approved the final version of the manuscript for submission.

## Results

### Demographic and clinical data of the mothers and their offspring at birth

Altogether, 45/327 (13.8%) mothers were carriers of genital HR-HPV, 47 (14.4%) had HR-HPV DNA in their baseline oral samples, whereas LR-HPVs were detected in both genital and oral samples of 8 (2.4%) mothers. Only 39 (11.9%) of the mothers had ASCUS or worse Pap-smear cytology. Altogether, 144 (44%) were current or past smokers, and 84.2% had started smoking before the age of 17 years. The majority (55.9%) had their sexual debut between 14–16 years of age. Seventy one mothers (21.7%) reported only 1–2 life-time sexual partners, while 60 (18.3%) had more than 10. Of the users of oral contraceptives (OC), 40.8% had initiated their use between 14–16 years of age, while 24 (7.3%) had never used OC. Altogether, 266/327 (81.3%) mothers reported no history of STI, while 80 (24.5%) mothers reported ever having genital warts. History of oral warts was rare (eight cases only), but skin warts were common (n = 166; 50.8%).

None of the mothers included in the FFHPV cohort had received the HPV vaccine. At baseline, the median age of the mothers-to-come was 26 years, (range 18–46), with a median gestational age of 40 weeks (range 31.6–42.5). Of all deliveries, 255 (77.6%) were vaginal and 74 (22.4%) caesarean (i.e. 327 mothers delivered 329 babies of whom two were twins). Altogether, 58% of the infants were first-born; 155 (48%) boys and 172 (52%) girls (two missing data in the record). Their median birth weight was 3.580 g (range 1.750–4.950 g). The mean time from the membrane rupture to the start of delivery was 4.1 h (range 0–53 h).

### HPV-seroprevalence of the mothers

**Figure 1:**
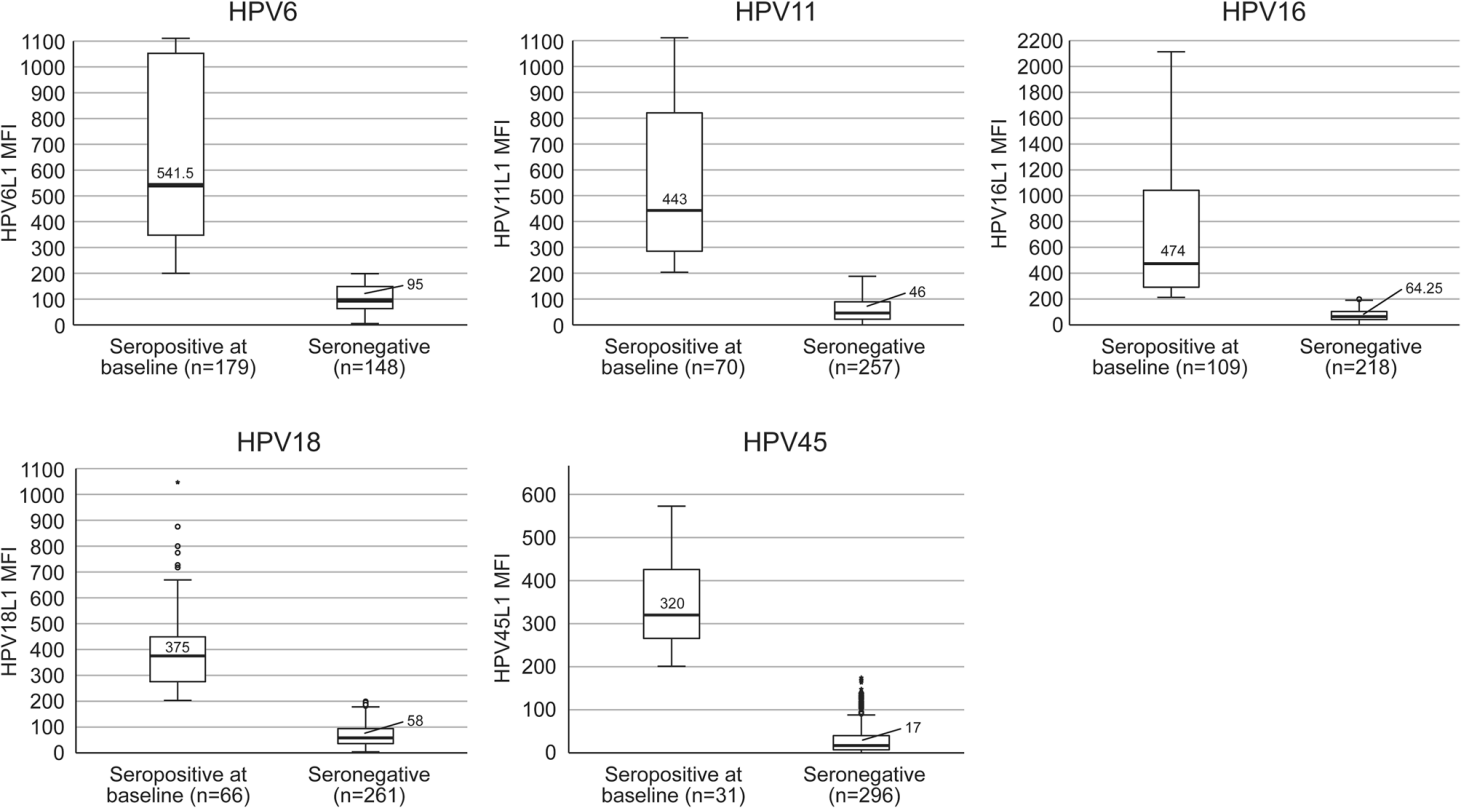
Mothers’ seropositivity at 3rd trimester by HPV type. HPV seropositivity to HPV6, HPV 11, HPV16, HPV18 and HPV45 in the pregnant mothers (n = 327) at their third trimester. The medium fluorescence (MFI) value of 200 or more was the cut-off for the seropositivity of the respect HPV types analyzed. The mean and the range of MFI in HPV-seropositive and HPV-seronegative mothers are given in the columns. None of the mothers had been vaccinated for HPV. *MFI* Mean fluorescence intensity, *n* number of the mothers stratified by HPV-seropositivity.

Figure 1 illustrates the mean IgG-antibody-levels to HPV 6/11/16/18/45 genotypes of the mothers at their 3rd trimester of pregnancy (baseline visit), classified as seropositive with 200 MFI cut-off (all HPV-types). HPV6-seropositivity was the most common (54.7%), followed by HPV16 (33.3%), HPV11 (21.4%), HPV18 (20.2%) and HPV45 (9.5%). The highest mean MFI-values were detected for HPV6 and HPV16 antibodies; 541 MFI and 474 MFI, respectively, with a range from 200 to over 1100 and 2100, respectively.

### HPV antibodies of the infants during their first 36 months

**Figure 2:**
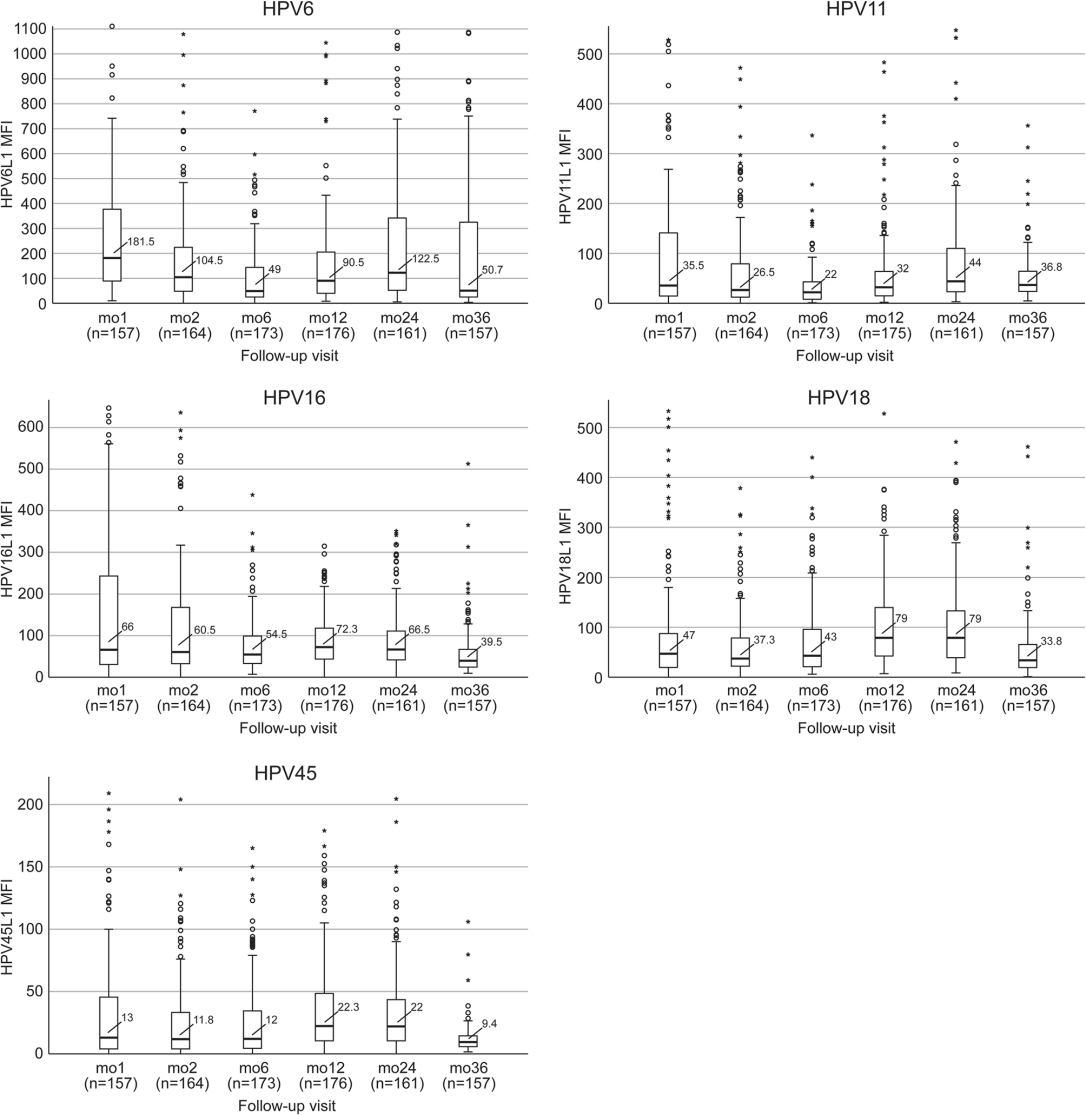
Antibody-levels to HPV6, HPV 11, HPV16, HPV18 and HPV45 in offspring born to seropositive mothers defined by MFI 200 or more for any HPV type. In total 179 of the 327 pregnant mothers were seropositive to HPV6, HPV 11, HPV16, HPV18 or HPV45 L1 protein (cut-off, any HPV type > 200 MFI). The mean HPV antibody levels and the range in their offspring are given as columns stratified by HPV serotypes and follow-up visits. The number of tested offspring (= n) of the seropositive mothers are given in parenthesis under the horizontal axis at the different follow-up visits (mo = month). Unfortunately, blood samples were not available from all infants at all different time points, and this is the reason why the numbers are not unanimous.

Figure 2 summarizes the median levels and range of HPV6/11/16/18/45 antibodies in the infants born to seropositive mothers (n = 179; any HPV type > 200 MFI). All infants born to the mothers so defined had measurable IgG- antibodies to the respective maternal HPV-serotype at the age of 1 month. However, only part of these infants had HPV6/11/16/18/45 antibody levels that exceeded the 200MFI cut-off for seropositivity: 45.8%, 18.4%, 30.5%, 12.1% and 5.7%, respectively. At the age of 1 month, the highest MFI mean value was found for HPV6 (MFI 181), followed by HPV16 (MFI 66), and HPV18, HPV11 and HPV45, all showing substantial variation (Fig. 2).

During the first 6 months, HPV6 and 11 antibody levels waned but started increasing again until the 24-month FU-visit. Similarly, the antibody-levels of the HR-HPVs remained relatively stable during the first 6 months and started to increase at the 12- and 24-month FU-visits followed by a decline at the 36-month FU visit.

**Figure 3:**
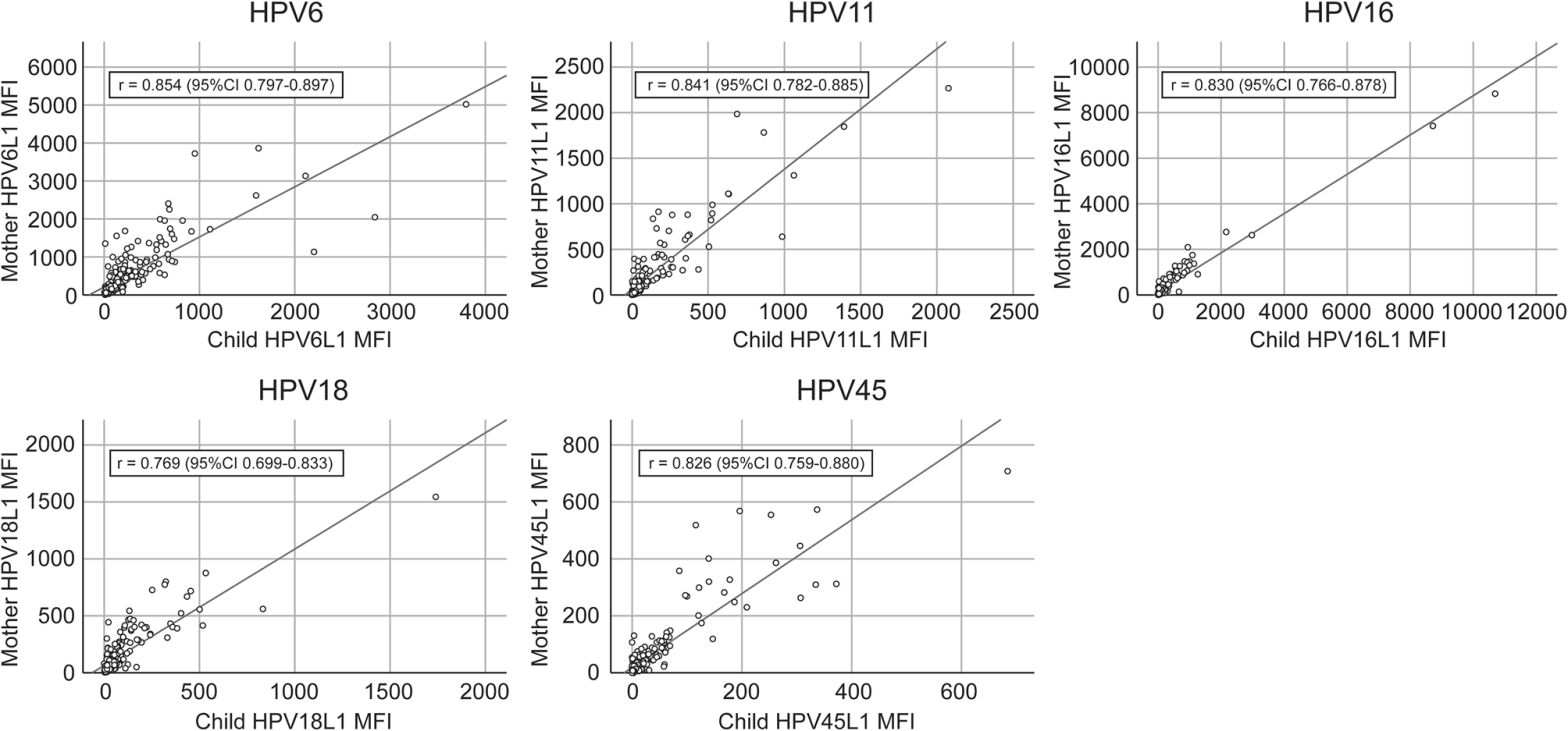
Correlation of antibody levels between the mothers—offspring pairs by HPV type. Correlation of individual HPV L1 serum antibody levels (MFI values) between pregnant mothers at 3rd trimester and offspring aged 1 month. None of the mothers had been vaccinated for HPV. Lines represent linear regression analyses; *r* Spearman coefficient, *MFI* Mean fluorescence intensity, *CI* confidence interval.

Mothers’ baseline HPV antibody levels had an almost perfect correlation with the respective antibody levels of their offspring at the 1-month FU, Spearman correlation coefficient (r) varying between 0.769 (HPV18) and 0.854 (HPV6) (Fig. 3). This correlation to infants’ serology remained highly significant (p < 0.001) for all HPV types at the 6-month FU (p < 0.001): HPV6 (r = 0.266), HPV11 (r = 0.344), HPV16 (r = 0.321), HPV18 (r = 0.220) and HPV45 (r = 0.259). At the age of 12 months, this correlation still persisted for HPV11 (r = 0.214, p < 0.001), HPV18 (r = 0.132, p = 0.030) and HPV45 (r = 0.169, p = 0.005) antibodies, whereas the infants’ HPV6 (r = 0.070, p = 0.250) and HPV16 antibodies (r = 0.100, p = 0.100) had lost their significant correlation with the baseline maternal antibody levels.

### HPV-seroprevalence of the offspring born to HPV-seropositive mothers

Table 1 summarizes the HPV type-specific seroprevalence and median MFI antibody-levels of seropositive infants born to mothers who were seropositive (≥ 200 MFI) to the same HPV-genotypes. Seroprevalence of the children is given as the number and (percentage) of seropositive (≥ 200MFI) offspring out of total number of tested offspring at each time point. At the age of 1 month, 40 to 62% of the infants were seropositive to any of the tested HPV-genotypes. During the FU, HPV6-seropositive children far outnumbered the children seropositive to the other HPV-genotypes. Infants’ HPV-seropositivity was at lowest at the 6-month FU-visit for all HPV types: 19.3%, 3.8%, 9.8% and 14.3% for HPV6, HPV11, HPV16 and HPV18, respectively, and seropositivity to HPV45 was non-existent from this point on. At the age of 2 years, 38.6% of the children were still seropositive for HPV6, and 14.9% were positive for HPV16 and HPV18, each. At the age of 3 years, 43 children were still HPV6-seropositive while only four and one were seropositive to HPV16 and HPV18, respectively.

**Table 1:** Concordance of HPV type-specific (HPV6, 11, 16, 18, 45) seropositivity between the mothers and their offspring.

Age of the infant	Offspring testing seropositive to the same HPV type as their mothers*				
	HPV6 n = 179*	HPV11 n = 70*	HPV16 n = 109*	HPV18 n = 66*	HPV45 n = 31*
	^a^Number and (percentage) of seropositive offspring / total number of tested offspring at each time point ^b^Median MFI (range)				
One month	72/134 (53.7)^a^ 606 (201–3798)^b^	29/53 (54.7) 530 (203–2076)	47/76 (61.8) 538 (201–10,695)	19/47 (40.4) 455 (212–1740)	9/21 (42.9) 341 (209–684)
Two months	42/132 (31.8) 547(202–2023)	17/51 (33.3) 267 (207–1470)	31/86 (36.0) 518 (206–6698)	12/54 (22.2) 273 (207–1094)	4/23 (17.4) 290 (204–446)
Six months	27/140 (19.3) 319 (209–4180)	2/53 (3.8) 287 (238–337)	9/92 (9.8) 269 (207–1577)	8/56 (14.3) 277 (200–401)	0/24
12 months	37/145 (25.5) 850 (201–3167)	5/56 (8.9) 312 (207–585)	10/89 (11.2) 232 (208–255)	13/55 (23.6) 290 (210–652)	0/22
24 months	51/132 (38.6) 877 (207–3110)	4/49 (8.2) 312 (207–585)	13/87(14.9) 420 (201–13,799)	7/47 (14.9) 279(209–1125)	0/22
36 months	43/128 (33.6) 692 (205–2373)	0/47	4/83 (4.8) 2 (203–365)	1/46 (2.2) 1225	0/19

The seropositivity cut-off in the mother–child pairs ≥ 200 MFI.

*Numbers of mothers seropositive to HPV6 (n = 179), HPV11 (n = 70), HPV16 (n = 109), HPV18 (n = 66) and HPV45 (n = 31).

^a^Numbers and (%) of the offspring testing seropositive to the same HPV type as their mothers/the total number of offspring tested at each time point. As an example of HPV6, 179 mothers (out of 327) tested seropositive to HPV6, but only 134 offspring of these mothers were serologically tested at 1-month; 132 at 2-month, 140 at 6-month, 145 at 12-month, 132 at 24-month and 128 at 36-month follow-up visit. The other HPV types follow the same pattern of presentation.

^b^Median MFI values and (range).

Altogether, 113 mothers had antibodies to more than a single HPV-type (≥ MFI 200). In total, 42 children born to these 113 mothers had also HPV-antibodies to multiple types. As a reference, there were 105 mothers classified seropositive to a single HPV-type only. Interestingly, only one offspring born to these “single-type seropositive” mothers had antibodies to multiple HPV-types.

### HPV-seroprevalence of the offspring born to HPV-seronegative mothers

To identify the infants’ seroconversion caused by an acquired HPV-infection during the 3-year FU, we evaluated the infants born to HPV-seronegative (< 200 MFI) mothers. Altogether, 53% (n = 58) of the infants born to 109 HPV-seronegative mothers did seroconvert to HPV by the age of 3 years, peaking at the age of 24 months (Table 2). HPV6-seropositivity was most prevalent among the children aged 1 year, and HPV6-seroconversion rate increased by age. Similarly, the mean levels of HPV6 antibodies were higher (> 1100 MFI at 12 and 24 months; 642 MFI at 36 months) than found for any other tested HPV-types. At the age of 3 years, 23 (4.7%) HPV-seropositive children were identified of whom six were seropositive to HPV6 or HPV18 at all three FU visits.

**Table 2:** Human papillomavirus (HPV) seroprevalence to HPV-genotype 6, 11, 16, 18 and 45 in the offspring at the age of 12-, 24, and 36 months born to the mothers seronegative for the respective HPV-genotype.

Follow up visit	12 months	24 months	36 months			
HPV antibodies in sera	Seropositive infants (n) mean MFI ± SD			Infants seropositive for the same HPV type at two visits (n)	Infants seropositive for the same HPV type at three visits (n)	Total number of HPV-seropositive offspring at the 12-, 24-, and 36-month FU-visits born to 109 HPV-seronegative mothers
HPV6	9 1152 ± 1033	10 1167 ± 954	16 642 ± 364	10	3	19
HPV11	7 498 ± 330	12 360 ± 150	3 293 ± 57	3		19
HPV16	3 276 ± 66	5 397 ± 252	1 549	3		6
HPV18	4 475 ± 260	14 417 ± 233	3 624 ± 445	2	3	13
HPV45	0	1 561	0			1
Total HPV-sero-positivity at the given age	23	42	23	18	6	In total, 53% (58/109)

The cut-off for seropositivity was ≥ 200 MFI.

Vaginal delivery was statistically significantly (p = 0.048), associated with infants’ seropositivity at the age of 2 years. The first-born children had significantly lower HPV6-antibody levels as compared with the non-first-born children at the age of 1 year (p = 0.0001) and 2 years (p = 0.023), as did HPV11-antibodies at 1 year (p = 0.0001).

### Outcome of HPV serology in children during the first 36 months of life

**Figure 4:**
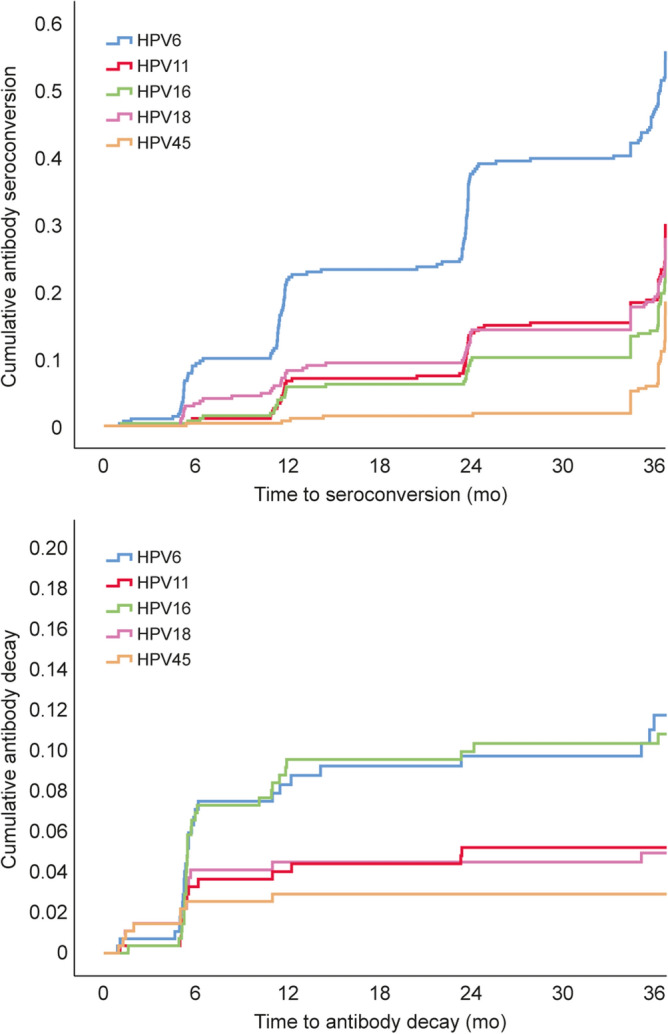
Cumulative seroconversion and antibody decay by HPV type in offspring during the first 3 years of life. Kaplan–Meier analysis showing 3 years cumulative antibody seroconversion (upper panel) and decay (lower panel) for HPV6, HPV 11, HPV16, HPV18 and HPV45 antibodies.

In Kaplan–Meier analysis for seroconversion (Fig. 4**,** upper panel), 46.1% of the infants showed seroconversion to HPV6, 14.1% converted to HPV11-, 10.3% to HPV16-, 13.8% to HPV18- and 1.6% to HPV45- L1-antigens (p = 0.0001). The mean (95% CI) seroconversion times for HPV6/11/16/18/45 were 18.7 months (16.8–20.6), 17.9 months (15.5–20.2), 17.3 months (13.7–20.8), 15.5 months (12.5–18.4), and 13.4 months (5.0–21.8), respectively (p = 0.639).

Figure 4 (lower panel) shows the Kaplan–Meier analysis for the cumulative decay of HPV antibodies in these infants without making any difference whether antibodies are of maternal origin or newly acquired by the infants. During the median FU-time, decay of HPV6/11/16/18/45-antibodies was observed in 15.5%, 5.2%, 10.6%, 5.2% and 2.6% of the infants, respectively (log-rank p = 0.0001). The mean (95% CI) times for antibody decay were as follows: 9.9 months (6.0–13.8), 8.5 months (4.5–12.5), 9.8 months (6.5–13.1), 9.1 months (2.3–15.9), and 4.0 months (1.2–6.8), for HPV6, HPV11, HPV16, HPV18 and HPV45, respectively (p = 0.054).

To explore the possible differences in HPV antibody decay or seroconversion between the infant antibodies of maternal origin and those newly acquired by the infants, we made a new Kaplan –Meier analysis of the infants stratified by their mother’s HPV-serostatus (HPV-seronegative or –positive). There was nearly no decay of the HPV-antibodies in infants born to HPV-seronegative mothers. The differences between infants born to seronegative and seropositive mothers was most obvious for HPV6 and HPV16 antibody decay. Due to the limited number of subjects, however, these differences were not statistically significant (HPV6 p < 0.195, HPV11 p < 0.995, HPV16 p < 0.334, HPV18 p < 0.417, HPV45 p < 0.267). Importantly, the serostatus of the mother for the individual HPV-types had no effect on infant’s seroconversion to the respective HPV-type.

### Predictors of the offspring’s seroconversion during the follow-up

Several maternal covariates such as age, Pap-smear cytology, smoking, age at the sexual depute, mother’s genital or oral warts, HPV DNA status at the 3rd trimester (Table 3) were included in the GEE-modelling in addition to pertinent covariates of the child to identify the predictors of the offspring’s seroconversion during the 3-year follow-up. In univariate GEE, mother’s seroconversion to LR-HPV predicted infant’s seroconversion to LR-HPV (p = 0.014; OR = 1.9, 95% CI 1.14–3.13) (Table 3). When adjusted for the other covariates, mother’s history of oral warts (p = 0.0001; OR = 55.9; 95% CI 6.74–463.08) and infant’s hand warts at the age of 3 years (p = 002; OR = 42.8, CI 4.15–440.76) were significant independent predictors of infant’s seroconversion to LR-HPV. In contrast, no significant predictors for infants’ seroconversion to HR-HPVs were disclosed by either univariate or multivariate GEE.

**Table 3:** Predictors of seroconversion of the infants to LR-HPV (HPV 6, 11) and HR-HPV (HPV 16, 18, 45) L1 antigens during the follow-up in time-dependent GEE modelling^1^ run in univariate model and as adjusted for other covariates.

Covariates	Seroconversion to LR-HPV (HPV6 L1 and/or HPV 11L1)*						Seroconversion to HR-HPV (HPV 16L1, HPV 18L1 and/or HPV 45L1)					
	Crude OR	95%CI	Significance	**Adjusted OR	95%CI	Significance	Crude OR	95%CI	Significance	@Adjusted OR	95%CI	Significance
Age (categorical) of mother	1.2	0.54–2.70	0.650	0.2	0.02–2.36	0.196	2.6	0.91–7.42	0.074	1.6	0.27–9.61	0.609
Genital HR-HPV DNA status of mother^2^	0.7	0.46–1.09	0.121	1.0	0.98–1.00	0.082	1.2	0.74–2.07	0.421	1.2	0.54–2.58	0.686
Oral HR-HPV DNA status of mother^2^	1.1	0.84–1.55	0.413	1.0	1.00–1.00	0.996	1.2	0.82–1.69	0.371	1.1	0.66–1.98	0.640
Baseline Pap cytology of mother (ASCUS cut-off)	0.9	0.60–1.41	0.693	1.0	0.99–1.00	0.428	1.3	0.81–2.22	0.249	1.5	0.62–3.59	0.366
Seroconversion to LR-HPV of mother (no conversion; ref)	**1.9**	**1.14–3.13**	**0.014**	1.8	0.60–5.24	0.301	1.7	0.96–3.00	0.066	1.3	0.53–2.94	0.607
Smoking status of mother	1.1	0.79–1.40	0.741	0.9	0.36–2.31	0.842	1.2	0.68–1.99	0.585	1.1	0.51–2.47	0.769
Mother’s history of genital warts (no history; ref)	1.2	0.72–2.12	0.438	0.5	0.17–1.42	0.188	0.7	0.40–1.27	0.245	0.5	0.20–1.08	0.074
Mother’s history of oral warts (no history; ref)	7.5	0.88–63.23	0.065	**55.9**	**6.74–463.08**	**0.0001**	1.1	0.20–5.66	0.943	1.3	0.14–11.75	0.818
Mother’s history of skin warts (no history; ref)	1.2	0.89–1.54	0.253	1.3	0.89–2.05	0.162	0.8	0.57–1.09	0.154	0.9	0.60–1.29	0.500
Sex of infant	1.0	0.62–1.54	0.910	0.7	0.27–1.67	0.389	1.1	0.63–1.78	0.816	1.0	0.47–2.27	0.927
Mode of delivery	1.0	0.57–1.85	0.927	2.4	0.73–7.91	0.148	0.7	0.32–1.41	0.324	0.6	0.20–1.59	0.280
Genital HR-HPV DNA status of infant^2^	0.8	0.61–1.05	0.112	1.0	0.99–1.00	0.482	0.9	0.66–1.19	0.418	0.9	0.53–1.54	0.706
Oral HR-HPV DNA status of infant^2^	1.0	0.75–1.27	0.844	1.0	1.00–1.01	0.140	0.9	0.71–1.23	0.644	0.8	0.46–1.57	0.602
Atopia	1.1	0.57–2.14	0.769	0.9	0.63–1.33	0.630	0.6	0.30–1.24	0.172	NCF	NCF	NCF
Allergy	1.0	0.77–1.29	0.985	1.4	0.53–3.73	0.500	0.7	0.46–1.12	0.149	NCF	NCF	NCF
Oral warts at 36 months	NC	NC	NC	NC	NC	NC	NC	NC	NC	NCF	NCF	NCF
Genital warts at 36 months	NC	NC	NC	NC	NC	NC	NC	NC	NC	NCF	NCF	NCF
Hand warts at 36 months	5.4	0.61–47.33	0.128	**42.8**	**4.15–440.76**	**0.002**	2.6	0.51–13.50	0.247	NCF	NCF	NCF

Statistically significant values are marked as bolded.

*Binary outcome (Conversion/No conversion) detected during the 36-mo follow-up (Cut-off 200 MFI, and > 2 × increase of Ab titers in two subsequent samples); ^1^Results obtained from time-dependent GEE with logit link for binary outcomes clustered by child ID number; **adjusted for all other covariates in the model; ^2^High-Risk (HR)-HPV detected at any follow-up (FU) visit;

OR, odds ratio; CI, confidence interval; NC, not computable, no relevant cases; NCF, not computable due to failure to achieve convergence.

## Discussion

This is the largest prospective study examining HR- and LR–HPV-serology in infants during the first 3 years of life. Clearly, IgG antibodies to the major capsid protein (L1) of HPV6, HPV11, HPV16, HPV18 and HPV45 are vertically transferred from the mother to her offspring, and concordance between maternal and newborn HPV-antibody levels remains high during the first 6 months after delivery. Importantly, HPV-seroconversion among children born to HPV-seronegative mothers is common.

Multiplex serology assay used in our study (also known as GST-L1 assay) is a commonly used method validated in several seroepidemiological studies on HPV, and thus appropriate for the present study. As determined from the natural history studies on HPV infection, GST-L1 seems to be s a good measure of cumulative immunity induced by the natural HPV infection but not a reliable marker of immune protection, because the assay does not distinguish between neutralizing and non-neutralizing antibodies^17^. HPV vaccine immunogenicity has been typically assessed by three different methods: (1) the virus like particle (VLP)-based enzyme-linked immunosorbent assay (VLP-ELISA), (2) competitive Luminex immunoassay (cLIA), and (3) secreted alkaline phosphatase-based pseudovirus neutralization assay (SEAPNA)^18^. The comparison between these four different assays is problematic as they do not measure equivalent aspects of the immune response and their seropositivity cut-offs are not calibrated against each other. Much of the observed lack of agreement can be explained by differences between the seropositivity cut-offs used by the different assays. The results of the available assays generally correlate well particularly in specimens with high antibody levels^18,19^.

Our results are in line with the few previous studies, even if performed with different assays, confirming close HPV-type-specific concordance between maternal and newborn antibodies, irrespective of the methods used for (i) newborn blood sampling (cord blood, dried blood spot or serum) and/or (ii) for HPV-antibody testing^7,8,11,17^. Important to note is that the mothers included in the prior studies referred here are non-HPV vaccinated, similarly as the mothers of our Finnish Family HPV Study cohort. Kawana and coworkers were the first to report the presence of neutralizing antibodies against HPV6 in sera of the infants born to mothers (n = 2) with HPV6-positive genital warts^7^. Maternal sera at delivery and at 5-week postpartum contained IgG- and IgM-antibodies reactive to HPV6L1/L2 capsids. The titers of the neutralizing HPV6 IgG-antibodies in cord sera were equivalent to those of maternal sera but no IgM-antibodies were detectable^7^.

Heim and coworkers analyzed HPV-antibody-levels in 104 mother-newborn-pairs using VLPs as the antigen^8^. Maternal IgG-positivity rates to HPV6, HPV11, HPV16, HPV18 and HPV31 in the newborn sera were 23.1%, 2.9%, 8.7%, 5.8%, and 9.6%, respectively^8^. IgM-HPV-antibodies were detected in 19% of the mothers, but not in newborns, except three who showed a weak IgM-immunoreactivity for HPV11 and HPV16 without any HPV IgG-antibodies. Similarly, Smith and coworkers analyzed 333 mother-newborn pairs reporting very high type-specific concordance for HPV16, HPV18, HPV31 and HPV33 antibodies, in the range of 96–97%^20^. Our results confirmed that the concordance of HPV-type-specific antibodies in mother-infant pairs remained high at least for 6 months. All infants born to HPV-seropositive mothers had measurable HPV-antibody levels at the age of 1 month, but these HPV-antibody levels did not reach the cut-off level of HPV-seropositivity (≥ 200 MFI) in around 50% of the tested infants (range from 38 to 60% for HPV16 and HPV18, respectively as given in Table 1).). This indicates a gradual waning of the HPV-antibodies starting at the age of 1 month and continuing until the age of 6 months.

Recently, Zahreddine and coworkers detected maternal antibodies in 58 newborns, of whom 23 were followed for their HPV-antibody dynamics during the first 24 months of life^11^. As might be expected, four mothers who had been vaccinated against HPV6/11/16/18 had the highest antibody levels, as did their offspring. These data corroborate with the recent two studies on maternal transfer of HPV-antibodies into the cord blood after vaccination with the 4-valent or 9-valent HPV vaccines^21,22^. These authors also described that newborns seropositive at birth cleared their HPV-antibodies (below the 200 MFI cut-off) in 80% and 100% by the 12- and 24-month FU, respectively^11^. However, measurable HPV-antibody levels (< 200 MFI) for HPV6/11/16/18 were detectable still in 24 and 15 children aged 1 and 2 years, respectively^11^. The significance of these low-titer HPV-antibodies, also found in the present study, remains unknown. In their series, the median clearance time for HPV6 antibodies was 6 months while antibodies to HPV11, HPV16 and HPV18 all cleared within 12 months^11^, similar as observed in the present study (Fig. 4). Our larger cohort and longer FU time might explain the minor dissimilarities (for HPV6 and HPV16) between these two studies.

Several studies on other pathogens highlight that maternal antibodies can be vertically transferred to the fetus and subsequently protect the neonate against the respective infection^23^. Maternal (IgG) immunoglobulins are transported across the placental membranes during pregnancy by an active, FcRn receptor-mediated process^23^. With the continuation of pregnancy, FcRn expression and trans-placental transport increase, peaking during the last 4 weeks of gestation. Thus, the 3rd trimester of pregnancy may serve as the optimal time to test for pathogen-specific antibodies. High maternal antibody levels are the most predictive factors of trans-placental antibody-transfer and maternal antibody concentrations in the infant^23^. Our results showed that nearly half of the infants born to seropositive mothers had HPV-antibody levels below the 200 MFI cut-off, while some had 10–80 times higher levels. This clearly suggests that there is a difference in the efficacy of the maternal antibody transfer related to maternal antibody concentrations^24^. Further studies are needed to understand the role of vertically transferred HPV-antibodies in protecting the infant from maternal HPV-infections either before birth or at early infancy.

Importantly, our results indicate that seroconversion to LR- and/or HR-HPVL1 proteins is possible already early in life, confirming the results of three previous studies^9,25,26^. In 1999, Mann and coworkers showed that 3/100 newborns developed HPV16-antibodies to HPV16 VLP at the age of 1 to 2 years. Two of these three children had HPV16-antibodies still detectable at the age of 5 years^25^. Af Geijersstam and coworkers measured serum antibodies to HPV16, HPV18 and HPV33 capsids (L1/L2 proteins) among 1031 children aged between 0–13 years^26^. In the age group of > 1.5–3 years (n = 181), seroprevalence for HPV16, HPV18 and HPV33 was 2.8%, 0.6% and 1.7%, respectively^26^. Mariais et al. (2007), however, reported higher seroprevalence rates in 37 children younger than 2 years: 5.4% to HPV16, 13.5% to HPV11, and 10.8% to HPV18 VLPs^9^. These data have an almost perfect match with the present results indicating that children born to HPV-seronegative mothers were seropositive to HPV16 (5.5%), HPV11 (17.4%) and HPV18L1 (11.9%) at the age of 1 to 3 years. In addition, Zahreddine and coworkers identified one child, HPV6/11-seronegative at birth, but seropositive with increasing antibody levels at 12- and 24-month FU-visits^11^.

Confirmed HPV-seroconversion during the first 3 years of life implicates an early exposure to HPV, i.e., a virus-producing infection capable of eliciting an immunological (antibody) response. However, we do not know asyet why some of the infants experience HPV seroconversion during these early months of their life. The two key unsolved questions are, (1) by which route has the exposure to different HPV-types taken place at early infancy? and (2) at which site has the serological response been mounted? The present study is the first to explore in more detail the predictors of HPV seroconversion at early age. The disclosed predictors (Table 3) suggest that HPV infection resulting in seroconversion at early childhood might be intra-familial, the mother being the most likely transmitter. This notion is supported by the discovery that mother’s history of oral warts (usually HPV6/11) and mother´s seroconversion to LR-HPVs are significantly associated with the offspring’s seroconversion to LR-HPV. Furthermore, we have previously demonstrated that persistent oral HR-HPV infection in children was associated with oral HPV-carriage of the mother at delivery as well as with the seroconversion of the mother to HR-HPV during the follow-up (OR = 1.60–1.92, 95% CI 1.02–2.74)^27^.

Taken together, the present results confirm that maternal IgG antibodies to HPVL1 ptotein are readily transferred to their offspring and remain detectable at least for 6 months, corroborating the known half-life of IgG-immunoglobulins. Furthermore, we also confirmed seroconversion to HPV-genotypes 6, 11, 16, 18 (but not HPV45) among children born to HPV-seronegative mothers, implicating an immune response to HPV-infection by these genotypes already at early infancy.

## Data Availability

Data and material are available from the corresponding author upon reasonable request.

## Code availability

Codes are available from the corresponding author upon reasonable request.
